# Eosinophilic esophagitis presenting with spontaneous esophageal rupture: a case report

**DOI:** 10.1186/s13256-019-2207-4

**Published:** 2019-09-03

**Authors:** Tanureet Kochar, Parminder Singh Dhingra, Muhammad Farhan Khaliq, Brittain Mcjunkin

**Affiliations:** 1 Department of Internal Medicine, West Virginia University Health Sciences Center, Charleston Division/Charleston Area Medical Center, 3110 MacCorkle Avenue SE, Room 3075, Charleston, WV 25304 USA; 2 Department of Internal Medicine, Mercy Saint Vincent Heath, Toledo, OH 43607 USA

**Keywords:** Eosinophilic esophagitis, Boerhaave’s syndrome, Esophagitis, Dysphagia

## Abstract

**Background:**

Eosinophilic esophagitis, once considered a rare disorder, has been increasingly recognized as a leading cause of dysphagia and food impaction in children and adults over the last few decades. It predominantly occurs in young men with a history of atopy. Dysphagia and food impaction are the most common presentations. However, rarely, spontaneous perforation (Boerhaave’s syndrome) may occur in association with eosinophilic esophagitis.

**Case presentation:**

A 40-year-old white woman with known history of eosinophilic esophagitis, who was non-compliant with treatment, presented with chest pain and developed acute spontaneous transmural esophageal perforation while eating a snack. Surgical repair was required.

**Conclusion:**

In a relatively young patient who presents with spontaneous esophageal perforation, eosinophilic esophagitis should always be ruled out as subsequent treatment may prevent recurrent perforation.

## Introduction

Eosinophilic esophagitis (EoE) was first described in the mid-1990s [1, 2]. With its increasingly recognized prevalence, EoE has emerged as one of the major causes of dysphagia and food impaction, primarily in young adults [3]. Prevalence has been estimated to be approximately 0.5 to 1/1000 persons in general populations and 40 to 90 cases/100,000 in the USA, with peak age range of 35 to 45 years [4]. EoE appears to be an allergen/immune-mediated disorder, perhaps related to changes in food allergens, increasing aeroallergens, or other factors [4]. EoE is a clinicopathological diagnosis requiring the presence of esophageal symptoms and eosinophils on histology. The most recent diagnostic guidelines [5] include the following: (1) clinical presentation suggestive of EoE (for example, dysphagia with endoscopic rings, furrows, stricture, crepe paper mucosa, concomitant atopic conditions, and so on); (2) esophageal eosinophilia with more than 15 eosinophils/high-power field; and (3) assessment of non-EoE disorders (including gastroesophageal reflux disease, hypereosinophilic syndrome, connective tissue disease, infection, drug hypersensitivity, or Crohn’s disease). Allergic and atopic conditions are seen in approximately 43% of the patients with EoE [6]. Whereas esophageal perforation following dilation in EoE has been well reported in the literature, spontaneous esophageal perforation following vomiting (Boerhaave’s syndrome) [7] has been rarely noted as a complication, yet can have catastrophic consequences.

## Case presentation

**Fig. 1:**
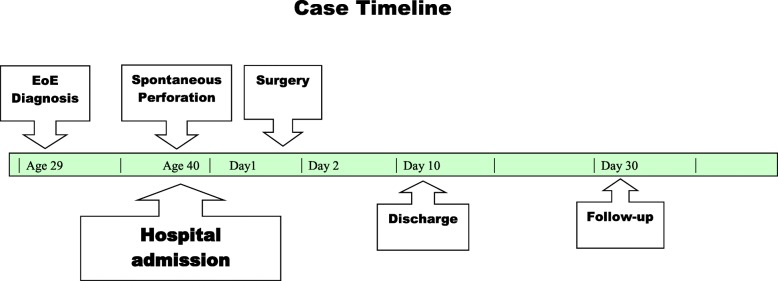
Case timeline. EoE eosinophilic esophagitis

**Fig. 2:**
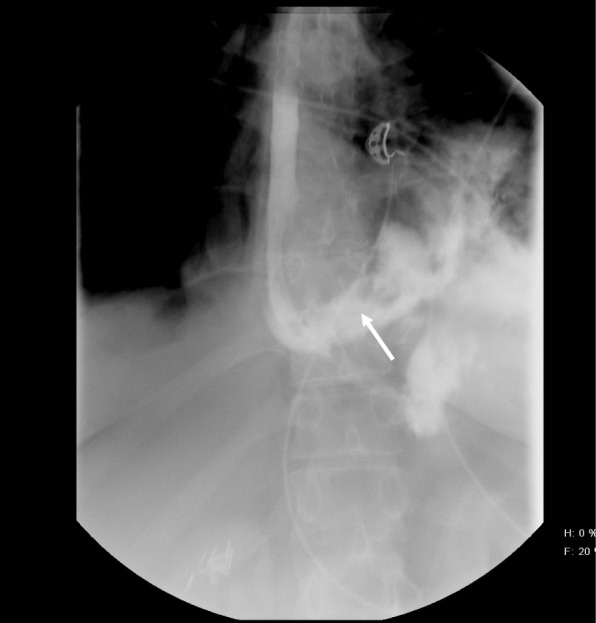
Esophagogram revealing large tear at gastroesophageal junction and extravasation of contrast into mediastinum (*arrow*)

**Fig. 3:**
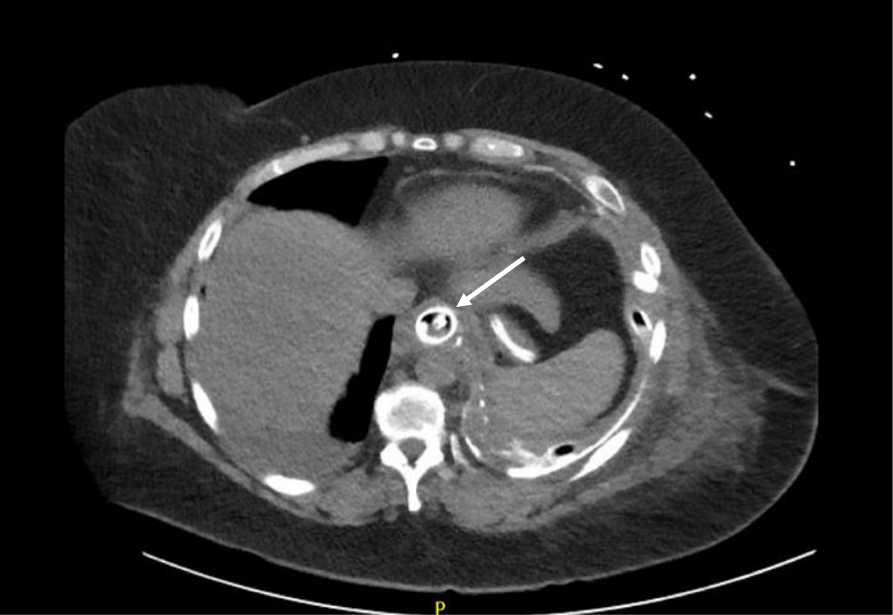
Computed tomography following surgery, showing esophageal stent (*arrow*) and residual contrast in the mediastinum

A 40-year-old white woman presented with an approximately 1-hour history of acute chest pain, which developed while eating a snack chip She induced vomiting to relieve the pressure, perceived a “popping” noise, and developed acute chest and epigastric pain. There had been no history of hematemesis, hemoptysis, melena, or weight loss. She had experienced episodic dysphagia since childhood and was diagnosed as having EoE at age 29. She had adhered poorly to local corticosteroid treatment and six-food elimination diet. She had experienced two previous perforations following dilation, which were treated conservatively. Endoscopies had revealed multiple fibrotic strictures and marked luminal narrowing. Initial eosinophil quantitation is unknown, but biopsies 4 years prior to current presentation showed 16 eosinophils per high-power field. Other past medical history included hypertension and obesity. She had no history of alcoholism, tobacco abuse, or illicit drug use. See timeline, Fig. 1. On presentation, following the onset of acute chest and upper abdominal pain, she was afebrile, restless, hypotensive with blood pressure (BP) of 88/69 mmHg, and tachycardic with heart rate greater than 120 beats per minute (bpm). Initial chest, heart, and lung examinations were normal. She had mild epigastric tenderness. Laboratory studies revealed white cell count of 33.8 × 10^9^/L with 90 neutrophils, hemoglobin of 14.3gm/dL, and creatinine 1.8 mg/dL (baseline creatinine 0.6 mg/dL). An esophagogram (Fig. 2) with Gastrografin (sodium diatrizoate and meglumine diatrizoate) showed a large tear in her esophagus at the gastroesophageal junction with leak of contrast within the left mediastinum. A chest X- ray revealed large left pleural effusion, with pneumomediastinum and an evident small pneumothorax. She developed respiratory distress requiring intubation and mechanical ventilation. She underwent emergency left thoracotomy, intercostal muscle flap placement over esophageal perforation, covered esophageal stent (Boston Scientific; WallFlex™) placement, and decortication of her left lung. Computed tomography (Fig. 3) following surgery showed intact stent and residual contrast in her mediastinum. Pleural fluid cultures grew *Enterobacter aerogenes* and *Candida*, which were treated with appropriate antimicrobials. Her diet was slowly advanced and when she was able to swallow a liquid diet, she was discharged home on day 10. She was seen on follow-up in the clinic and was doing well.

## Discussion

Spontaneous esophageal perforation with or without food impaction is one of the rare but life-threating complications of EoE. In 2008, Straumann *et al*. confirmed one case of spontaneous esophageal rupture among 251 patients with established EoE over an 18-year period [8]. In 2013, Jackson *et al.* reported a case series of four patients out of 447 with EoE over a 10-year period, who presented with Boerhaave’s syndrome in the absence of food impaction [9]. None of the four patients had an established diagnosis prior to presentation, and one patient had experienced a prior perforation [9]. Ten out of 13 previous case reports (including Straumann) were reviewed by Vernon *et al*. in 2014, and two more cases were included [10]. Fontillon and Lucendo described a case of non-traumatic Boerhaave’s syndrome requiring transhiatal esophagectomy [11]. Eosinophilic infiltration was demonstrated throughout the esophageal wall. Intense inflammation and fibrous remodeling was also appreciated [11]. Gunasekaran *et al.* in 2016 reported two cases of spontaneous esophageal perforation in children with previously undiagnosed EoE [12]. Runge *et al*. performed a retrospective review on 511 patients and documented six cases with spontaneous esophageal perforation [13]. Four other cases occurred after instrumentation [13]. Including our case, there appears to be a total of 28 reported cases of spontaneous perforation in EoE over the past few decades. In these cases, diagnosis of EoE was known in only three patients prior to perforation. Surgery was required in 13 of the 28 patients. There were no deaths.

In the review by Runge *et al*., longer duration of symptoms before diagnosis, prior history of food impaction, and the presence of a focal stricture were independent risk factors associated with perforation [13]. The progression of disease to fibrostenotic features was most commonly seen in adults and included fixed rings, dominant esophageal strictures, and narrow caliber esophagus. In patients with spontaneous perforation, the triad of vomiting, severe chest/epigastric pain, and pneumomediastinum was found [13]. It has been hypothesized that the variable clinical spectrum of EoE depends on the extent of infiltration of eosinophils in the wall of the esophagus. The presence of transmural extension of eosinophils leads to intense inflammation, fibrosis, and remodeling, affecting esophageal dispensability and integrity, probably predisposing to rupture [11, 12]. We note that our patient may not have had complete food impaction but developed esophageal rupture upon self-induced vomiting. Although pneumomediastinum has been reported in bulimia, only one case of esophageal rupture has been confirmed in these patients with no prior esophageal pathology [14], thus lending credence to inflammatory infiltration of EoE as a predisposing factor for esophageal perforation. In addition, this evident predisposition may be further substantiated by the history of recurrent spontaneous perforation in two patients with EoE. Importantly, we note that patients who presented with spontaneous perforation usually had undiagnosed EoE, only confirmed after the event. Subsequently, patients with established EoE who are compliant with medical treatment may be less likely to present with recurrent esophageal rupture. Our patient had been non-adherent to management approaches.

Patients with EoE with iatrogenic or spontaneous perforation require surgery if there is extensive extravasation of contrast into the mediastinum and toxic presentation, while others may be managed conservatively, possibly including stent placement [15].

## Conclusion

In a young patient with spontaneous esophageal rupture and no predisposing factors, such as binge alcoholism, EoE should be considered the most likely cause. Confirmation of diagnosis appears to be extremely important, as follow-up pharmacologic and/or dietary treatment may minimize future complications, including recurrent perforation.

## Data Availability

Data sharing is not applicable to this article as no datasets were generated or analyzed during the current study.
